# Direct Oral Anticoagulants as Successful Treatment of Heparin-Induced Thrombocytopenia: A Parisian Retrospective Case Series

**DOI:** 10.3389/fmed.2021.713649

**Published:** 2021-08-05

**Authors:** Julie Carré, Hippolyte Guérineau, Christine Le Beller, Laëtitia Mauge, Benoit Huynh, Roya Nili, Benjamin Planquette, Sylvain Clauser, David M. Smadja, Dominique Helley, Agnès Lillo-Le Louet, Nicolas Gendron, Leyla Calmette

**Affiliations:** ^1^Innovative Therapies in Haemostasis, INSERM, Université de Paris, Paris, France; ^2^Hematology Department, Assistance Publique Hôpitaux de Paris, Centre-Université de Paris, Paris, France; ^3^Hematology-Immunology-Transfusion Department, Hôpitaux Universitaires Paris Ile De France Ouest, Université Versailles Saint Quentin, Boulogne, France; ^4^Pharmacovigilance Department, Assistance Publique Hôpitaux de Paris, Centre-Université de Paris, Paris, France; ^5^INSERM UMR-S970, Centre de Recherche Cardiovasculaire de Paris, Paris, France; ^6^Hematology Department, Institut Mutualiste Montsouris, Paris, France; ^7^Respiratory Medicine Department and Biosurgical Research Lab (Carpentier Foundation), Assistance Publique Hôpitaux de Paris, Centre-Université de Paris, Paris, France; ^8^Hematology Department and Biosurgical Research Lab (Carpentier Foundation), Assistance Publique Hôpitaux de Paris, Centre-Université de Paris, Paris, France

**Keywords:** heparin-induced thrombocytopenia, direct oral anticoagulant, thrombosis, platelets, apixaban, rivaroxaban

## Abstract

**Background:** Heparin-induced thrombocytopenia (HIT) is a prothrombotic life-threatening disorder caused by an adverse reaction to heparin exposure. In this context, it is imperative to stop heparin immediately and to replace it by a non-heparin anticoagulant therapy. Despite their advantages, the use of direct oral anticoagulants (DOACs) is only emerging for HIT treatment, and their use remains rare.

**Objective:** To improve our knowledge on the emerging role of DOACs as treatment of HIT and give an overview of our local practices in this context.

**Patients/Methods:** This is a multi-centric retrospective case series of HIT patients referred to our Parisian pharmacovigilance network and treated with DOACs.

**Results:** We report the cases of seven patients from four healthcare centers, diagnosed with HIT (4T score ≥ 4, positive anti-PF4/heparin immunoassay and positive serotonin-release assay) and treated with DOACs. After a few days on substitutive parenteral treatment (*n* = 6) or directly at HIT diagnosis (*n* = 1), these patients were treated with either rivaroxaban (*n* = 6) or apixaban (*n* = 1) during acute HIT phase. Mean time to platelet count recovery after heparin discontinuation was 3.3 days (range 3–5). No patient experienced major or clinically relevant non-major bleeding or thrombosis that could be related to DOAC treatment during follow-up.

**Conclusions:** Our cases studies are consistent with recent guidelines credit to the potential and safe use of DOAC during acute HIT in clinically stable patients.

## Introduction

Heparin-induced thrombocytopenia (HIT) is a rare complication of heparin therapy that results from an immune mechanism mediated by immunoglobulin G (IgG) directed against platelet 4 factor/heparin (PF4/H) complexes (1–3). Heparin-induced thrombocytopenia represents a life-threatening situation because of its high paradoxical thrombotic risk. Indeed, activating monocytes, endothelial cells, platelets, and therefore coagulation, anti-PF4/H antibodies are responsible for arterial and venous thromboembolism (VTE) (1). Heparin-induced thrombocytopenia probability is estimated by the 4T score which considers various biological and clinical settings and stratify HIT risk as low (score 1–3), intermediate (score 4–5), or high (score 6–8) (4–6). Heparin discontinuation and its immediate replacement with a non-heparin anticoagulation when HIT probability is high or intermediate are part of the recommendations (5, 7). Alternative therapeutics usually recommended are parenteral and include danaparoid and argatroban (7, 8). Fondaparinux may be used as well (5). The management of these alternative parenteral drugs is often difficult, in particular in non-expert centers: they may require laboratory monitoring for dose adjustment, are costly and may expose the patient to a significant hemorrhagic risk (9), justifying a prolonged hospitalization. Direct oral anticoagulants (DOACs) have been available and have demonstrated non-inferiority to VKA in the treatment of acute symptomatic VTE with reduced risks of major bleeding (10, 11). Direct oral anticoagulants are taken orally, have a rapid onset of anticoagulation and need no routine monitoring. No cross reactivity with anti-PF4/H antibodies has been reported with DOACs (12) that may offer valuable alternative to current therapeutics in HIT management. Indeed, first or second line DOACs treatment showed promising results in isolated cases or small cohorts of HIT patients (13–17). Recently American Society of Hematology (ASH) suggested rivaroxaban as a treatment for HIT in non-life-threatening situations regardless of the presence of a thrombotic complication (7). Furthermore, proposals from the French Working Group on Perioperative Hemostasis (5) are quite similar to those of the ASH. Direct oral anticoagulants seem to be an attractive alternative therapy for HIT treatment but despite these expert recommendations, DOACs use still remains rare compared to well-established therapies in this setting (13).

To improve our knowledge on the emerging role of DOACs in HIT, we retrospectively analyzed HIT diagnosed patients treated with DOACs in our centers and aimed to assess the efficacy and safety of DOACs during HIT.

## Patients/Methods

### Study Population and Design

In this multi-centric retrospective case series, we investigated clinical and biological data of HIT patients referred to our local pharmacovigilance network, collected between January 1st, 2010 and June 30th, 2020 in a survey conducted by the pharmacovigilance center localized in the Hôpital Européen Georges Pompidou (HEGP, Assistance Publique-Hôpitaux de Paris, APHP, Paris, France) which provides, in collaboration with hematology department (forming the HIT team), management recommendations on suspected and/or diagnosed HIT patients from five other hospitals located in Paris region. The study was performed in accordance with the Declaration of Helsinki. The institutional review board of each center approved the study, and anonymous data collection was declared to the appropriate authorities (authorization protocol number: CNIL-1922081). Only HIT patients who received DOAC during HIT were included in the present study. No exclusion criteria were applied. For all patients included, baseline characteristics (age, gender, body mass index), clinical, biological, and HIT treatment-related data were retrieved from the medical records. Creatinine clearance (CrCl) was calculated using the Cockcroft and Gault formula.

### HIT Diagnosis Strategy

Heparin-induced thrombocytopenia diagnosis was supported by clinical and laboratory data. Criteria for HIT suspicion used by the HIT team were those of the 4T score (4, 7, 8). An intermediate or high probability for HIT (4T score ≥4) (4) led to IgG anti-PF4/H antibodies testing by enzyme-linked immunosorbent assay (Zymutest HIA IgG HYPHEN BioMed, Neuville sur Oise, France). As only a subset of anti-PF4/H antibodies is able to activate platelets and cause clinical HIT, a functional assay was required to confirm HIT diagnosis when antibody testing is positive (5) [optical density (OD), >0.5 was defined as clinical cut-off for positivity according to the manufacturer's instructions].

Thus, the ≪gold standard≫ 14C-serotonine-release assay (SRA) was performed. This test investigates the capability of the antibodies to activate platelets in the presence of heparin. Washed platelets of selected healthy donors free from aspirin and non-steroidal anti-inflammatory drugs for at least 10 days and known to react well in the SRA were used for the assays. Patient plasma and increasing concentrations of unfractionated heparin (UFH) were added to these healthy donor platelets previously incubated with radioactive 14C-serotonin. 14C-serotonin released by activated platelets in response to the presence of anti-PF4/H antibodies was measured. A positive SRA test was defined by significant serotonin release (>30%) from donor platelets when mixed with patient plasma and low heparin dose (0.1 U/ml) associated with inhibition of platelets activation by at least 50% from maximal activation values when high dose heparin (100 U/ml) is added (18). However, if the release was over 30% in the presence of high UFH concentration, the result was considered as uncertain. The result was considered negative if serotonin release was <20% and uncertain if it was between 20% and 30% using low UFH doses with at least three healthy platelet donors. Positive and negative control plasma were tested in parallel in each series. All assays were performed at HEGP in hematology department according to the recommendations of the International Society on Thrombosis and Hemostasis (ISTH) (19) and to local practice (20). All conclusions were reported in a medical letter kept in the patient's medical files.

### Statistical Analysis

Clinical and biological characteristics were expressed using standard proportions, median, and range (min–max values). To assess efficacy and safety of DOACs in HIT, we focused on the occurrence of thrombosis and bleeding events (major or clinically relevant non-major bleeding) according to ISTH criteria during DOACs therapy (21, 22). We reported clinical and biological outcomes, time under parenteral non-heparin anticoagulation, time to platelet recovery (i.e., time to reach platelets ≥150 × 10^9^/L after heparin discontinuation) and time before patient discharge.

## Results

**Figure 1:**
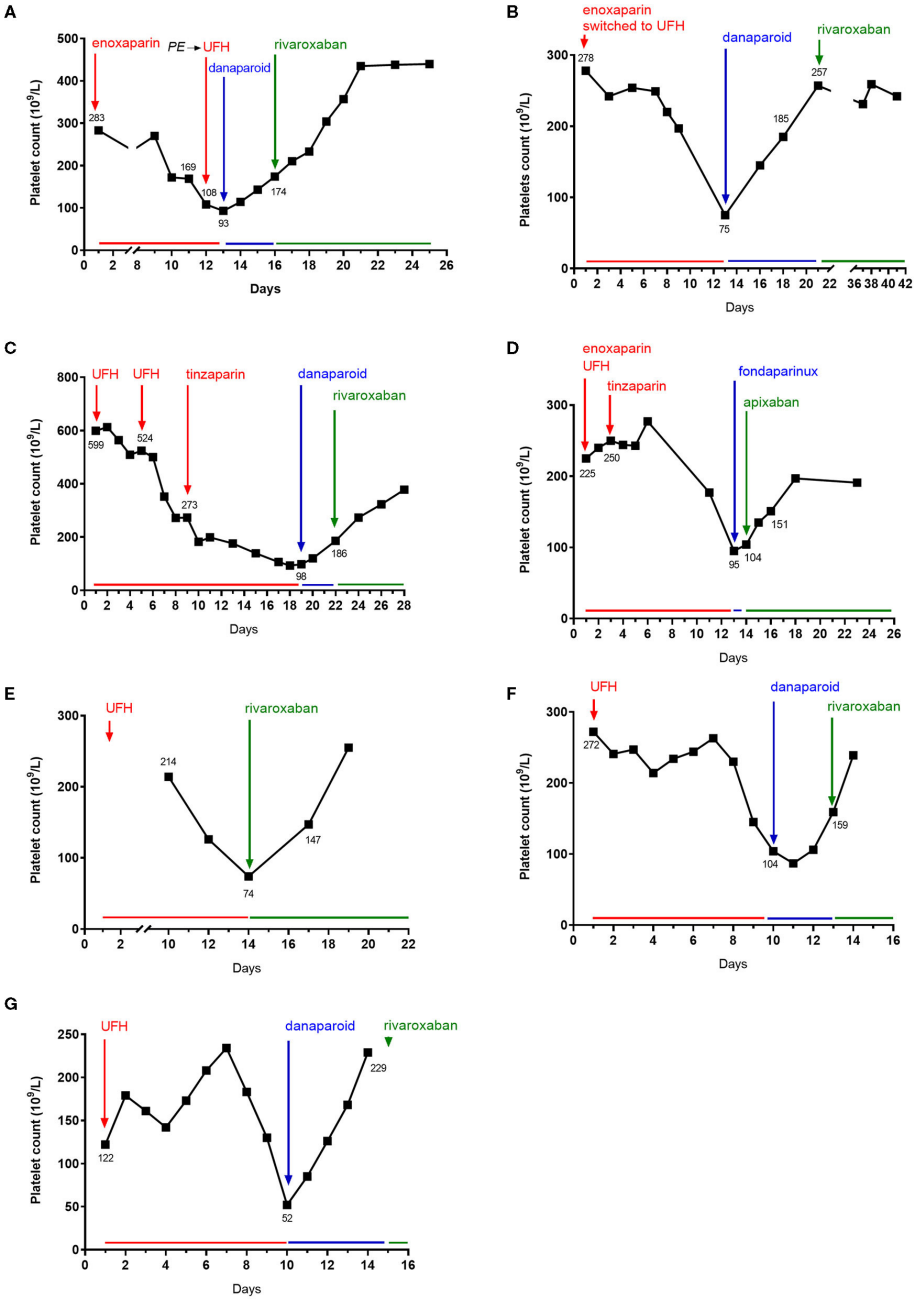
Platelet count evolution over time after heparin initiation. Day 1 as the first day unfractionated heparin (UFH) or low molecular weight heparin treatment (LMWH) administration. **(A–G)**, respectively, represent patients #1 to #7.

Between January 1st, 2010 and June 30th, 2020, 376 cases of HIT were diagnosed and referred to our local pharmacovigilance center. Among them, seven (1.9%) patients from four healthcare centers were treated by DOAC during acute or subacute HIT context as defined in ASH guidelines (7), between 2013 and 2020. There were four (57.1%) women and three (42.9%) men with a median age of 72 years (range 45–87). Heparin type administrated to patients was UFH only (*n* = 2, 28.6%), low molecular weight heparin (LMWH) only (*n* = 1, 14.3%) or both (*n* = 4, 57.1%), at prophylactic (*n* = 3) or therapeutic (*n* = 4) regimen. Clinical and biological characteristics of patients are described in Table 1. Patients displayed either an intermediate (*n* = 5, 71.4%) or a high (*n* = 2, 28.6%) 4T score probability for HIT. Heparin therapy was administered during a median time of 12 days (range 9–14) before HIT suspicion. The changes in platelet count are shown in Figure 1. At baseline (first day of heparin treatment), the median platelet count was 272 × 10^9^/L (range 122–599 × 10^9^/L) and at nadir 87 × 10^9^/L (range 52–95 × 10^9^/L). Heparin-induced thrombocytopenia-related thrombosis (HITT) occurred in only one patient, as pulmonary embolism (PE) while on prophylactic enoxaparin treatment.

**Table 1:** Characteristics of HIT-suspected patients treated by DOACs.

**Patient**	**#1**	**#2**	**#3**	**#4**	**#5**	**#6**	**#7**
Age (years)	45	87	85	72	73	48	66
Sex	F	F	F	M	M	F	M
BMI (kg/m^2^)	28	NA	NA	26	25	16	23
CrCl (ml/min)	171^*^	66	48	80	77	71	65
**Heparin Therapy**							
Indication	MI	PE	AF	PE	PE+DVT	Heart failure	Cardiac surgery
Type	Enoxaparin switched to UFH	Enoxaparin switched to UFH	UFH switched to tinzaparin	UFH switched to tinzaparin	Enoxaparin	UFH	UFH
Duration (days)	12	13	14	12	13	9	10
**HIT Diagnosis**							
4T score	6	5	4	4	6	4	5
HITT	PE	None	None	None	None	None	None
IgG PF4/H (OD)^a^	2.81	1.97	2.73	2.09	1.6	2.84	2.67
SRA (%)							
Low dose UFH	59	104	92	82	NA	91	89
High dose UFH	7	0	5	0	NA	4	14
Platelet count nadir (× 10^9^/L)	93	75	93	95	74	87	52

*F, female; M, male; NA, non-available; BMI, body mass index; CrCl, creatinine clearance evaluated with Cockcroft and Gault formula; MI, myocardial infarction; PE, pulmonary embolism; AF, atrial fibrillation; DVT, deep vein thrombosis; UFH, unfractionated heparin; HIT, heparin-induced thrombocytopenia; HITT, heparin-induced thrombocytopenia-related thrombosis; IgG, Immunoglobulin G; PF4/H, platelet factor 4/heparin; OD, optical density; SRA, serotonin release assay*.

* *Of note, as in obese patient, glomerular filtration rate calculated with MDRD formula is 127.5 ml/min/1.73 m^2^*.

a *Positive threshold OD > 0.5*.

Anti-PF4/H antibodies were positive in all patients (median OD 2.67, range 1.60–2.84). 14C-serotonine-release assay was positive in six tested patients confirming HIT diagnosis and was not performed for patient #5 (no serum or plasma sample available).

Management of HIT is described in Table 2. In five (71.4%) patients, heparin therapy was initially switched to parenteral danaparoid for a median duration of 4 days (range 3–8) before being switched to DOAC. Patient #4 received only one fondaparinux injection before switching to DOAC and patient #5 was directly treated with rivaroxaban.

**Table 2:** Management of HIT-suspected patients.

**Patient**	**#1**	**#2**	**#3**	**#4**	**#5**	**#6**	**#7**
**Parenteral Anticoagulant Switch After Stopping Heparin**							
Type, dose, duration	IV danaparoid bolus 3,750 U; then 300 U/h, 2 h; then 200 U/h, 4 days	IV danaparoid bolus 2,500 U; then SC 2,000 U BID, 8 days	IV danaparoid bolus 2,000 U then 300 U/h, 2 h; then 150 U/h, 3 days	SC fondaparinux 7.5 mg QD, 1 day	None	IV danaparoid 300 U/h, 3 days	IV danaparoid bolus 3,750 U; then 300 U/h, 2 h; then 200 U/h, 4 days
**DOAC Therapy**							
Platelet count at DOAC introduction (× 10^9^/L)	174	257	186	104	74	159	229
DOAC type, dose, duration	Rivaroxaban 15 mg BID for 21 days; then 20 mg QD ≥ 1 month	Rivaroxaban 15 mg BID for 32 days; then 20 mg QD ≥ 1 month	Rivaroxaban 15 mg BID for 21 days; then 20 mg QD ≥ 1 month	Apixaban 5 mg BID for 6 months	Rivaroxaban 15 mg BID for 21 days; then 20 mg QD ≥3 months	Rivaroxaban 20 mg QD for 1 month	Rivaroxaban 20 mg QD for 1 month
**Follow-Up After Heparin Discontinuation**							
Time to platelet recovery (days)	4	5	3	3	3	2	3
Outcome events^*^	None	None	None	None	DVT^**^	None	None
Time of last follow-up after DOAC introduction (months)	41	4	14	12	45	4	1

*IV, intravenous; SC, subcutaneous; U, units; DOAC, direct oral anticoagulant; BID, twice daily; QD, once daily*.

* *Thrombosis, major bleeding, clinically relevant non-major bleeding or death*.

** *Rivaroxaban withdrawal for 5 days for kidney surgery before completion of 1 month of treatment*.

Concerning DOAC therapy, six (85.7%) patients were treated with rivaroxaban, among which four were treated by 15 mg twice daily (BID) for 21 days or more followed by 20 mg once daily (QD) for more than 1 month. Two patients were directly treated with rivaroxaban 20 mg QD for 1 month. Only one patient (14.3%) received apixaban (5 mg BID for 6 months). Two patients were still thrombocytopenic (104 and 74 × 10^9^/L) at DOAC initiation, including patient #5 receiving rivaroxaban as initial substitutive therapy. All patients had normal renal function at the time of DOAC initiation. Median time to platelet recovery after heparin discontinuation was 3 days (range 3–5).

After DOAC initiation, patients #1 to #6 were hospitalized for a median time of 8.5 days until discharge (range 4–21). Patient #7 was transferred to a cardiac rehabilitation center at the date of DOAC initiation. Median platelet count at patient discharge was 242 × 10^9^/L (range 191–440 × 10^9^/L). No bleeding or thrombotic events occurred during hospitalization and under DOAC therapy, excepting patient #5 who presented a thrombosis recurrence with a lower limb deep venous thrombosis after stopping rivaroxaban treatment for 5 days in the first month of treatment, to undergo a kidney surgery in a neoplastic context without bridging anticoagulant treatment.

## Discussion

This retrospective case series shows that DOAC therapy used in first or second line was effective and well tolerated for the management of acute or subacute HIT. Platelet recovery was observed in each reported case and occurred in the same way in HIT patients receiving danaparoid or early DOAC treatment. Patients did not develop thrombotic events relative to DOAC inefficiency, and no bleeding events were reported during DOAC treatment. Rivaroxaban and apixaban are the most evaluated DOACs in the management of HIT in small retrospective series or case reports (23) but not in randomized clinical trials compared to danaparoid and/or argatroban.

In 2017, Kunk et al. showed platelet recovery and no new thrombosis event in 12 HIT-suspected patients who received DOAC (apixaban, *n* = 10, rivaroxaban, *n* = 2) following initial therapy with argatroban or bivalirudin for a mean time of 7 days until platelet count ≥50 × 10^9^/L (24). However, two patients presented major bleeding during follow-up probably linked to other causes (24). In a retrospective analysis, Davis et al. (14) described safety and efficacy of DOAC therapy in first line (apixaban, *n* = 5) or second line after argatroban initial treatment (apixaban, *n* = 4, rivaroxaban, *n* = 3) in twelve HIT-diagnosed patients. All patients showed platelet recovery and no thrombotic events or major bleeding were reported. To date, only Linkins et al. (16) performed a prospective study to evaluate rivaroxaban in twelve HIT-confirmed patients including six presenting HITT. Seven patients received fondaparinux or danaparoid for few days prior to rivaroxaban initiation. Platelet recovery was observed in nine patients among ten with initial thrombocytopenia (median time to recovery of 7 days) and one (8.3%) patient presented extensive VTE.

In our study, two patients were thrombocytopenic when DOAC was introduced, including the one with DOAC as initial alternative therapy, who remained free of thrombosis or bleeding events until the end of follow-up, as previously described by Warkentin et al. (23) (9/16 DOACs treated patients, with or without previous parenteral treatment, while still thrombocytopenic).

Rivaroxaban and apixaban showed convincing preliminary results used as first or second line of HIT treatment (23). Among our case series, 85.7% patients were treated with rivaroxaban. Efficacy and safety of rivaroxaban used as first line HIT treatment, defined by platelet recovery, no recurrent thrombosis and no major bleeding event, were also demonstrated in a retrospective series of nine patients presenting HITT only documented by 4T score and positive IgG-specific antiPF4/H (17). Moreover, Warkentin et al. (23) reported the cases of three HITT patients treated with rivaroxaban as first line treatment and one HITT patient who has been treated by fondaparinux for 4 days before being switched to rivaroxaban. The four patients were thrombocytopenic at DOAC introduction and authors report no thrombotic or bleeding events. Only one patient experimented HITT in our case series, but she was treated with rivaroxaban after 4 days of danaparoid and after platelet recovery.

Since 2018, ASH guidelines (7) recommend DOACs as first line HIT treatment for patients without vital or functional risk. Concerning the choice of DOAC, most of the published experience in HIT is with rivaroxaban and ASH guidelines suggest in acute HIT, rivaroxaban 15 mg BID is required until platelet recovery, followed by 20 mg QD if ongoing anticoagulation is needed. In acute HITT patient, rivaroxaban 15 mg BID should be maintained for 21 days followed by 20 mg QD for at least 3 months.

In the present study, five patients received rivaroxaban preceded by a short course of danaparoid. Unlike the switch from an initial treatment by argatroban and fondaparinux to DOAC, switch from initial danaparoid to DOAC has been much less reported in publications. Switching from danaparoid to DOAC is complex due to the lack of appropriate guidelines and the long half-life of danaparoid. In the cases reported here, starting rivaroxaban at the time of next scheduled danaparoid subcutaneous injection or once danaparoid anti-Xa activity is under 0.5 U/ml seems a safe and effective approach in managing acute HIT. Nevertheless, four patients (#1, #2, #3, and #6) could also have been treated with first line rivaroxaban according to ASH guidelines. Moreover, rivaroxaban 15 mg BID was started after platelet recovery to decrease the thrombotic risk for patients #2 and #3 despite the absence of HITT, although in these cases, a dose of 20 mg QD could have been directly used as proposed by ASH guidelines and as applied for patient #7. Patient #2 presented recent PE (<1 month) that could have justified an initial dose of 15 mg BID in the absence of guidelines regarding rivaroxaban use in case of switch from injectable anticoagulant therapy in the treatment of venous thrombosis.

Our study has several limitations. First, because HIT is rare, we could only evaluate seven patients treated by DOACs during the study period. Second, HIT suspicion in patient #5 could not be confirmed with SRA, but a 4T score of six with positive IgG anti-PF4/H and platelet recovery following heparin interruption were strong elements in favor of HIT diagnosis. In addition, the long-term outcomes of patients could not be assessed. Third, we acknowledge that we report the cases of HIT stable patients: six in medical wards and only one in surgical context. Moreover, only one patient had HITT. As suggested by recent systematic reviews and meta-analyses, DOAC use in HIT may be biased, selecting a subgroup of HIT patients with a particularly favorable prognosis (25).

The present study brings supplemental arguments to support the use of DOACs in first or second line to treat acute or subacute HIT in stable patients. Nevertheless, further larger and randomized controlled studies evaluating DOAC therapy in HIT-confirmed patients are needed to confirm these findings, and especially in HITT and/or less stable patients.

## Data Availability Statement

The original contributions presented in the study are included in the article/supplementary material, further inquiries can be directed to the corresponding author/s.

## Ethics Statement

The studies involving human participants were reviewed and approved by The study was performed in accordance with the Declaration of Helsinki. The institutional review board of each center approved the study and anonymous data collection was declared to the appropriate authorities (authorization protocol number: CNIL-1922081). Written informed consent for participation was not required for this study in accordance with the national legislation and the institutional requirements.

## Author Contributions

JC, LC, and NG collected data, analyzed data, and wrote the manuscript. HG, CL, AL-LL, and BH contributed to data collection. CL, AL-LL, LM, BP, SC, DS, DH, and RN contributed to data analysis and manuscript revision. All authors contributed to the article and approved the submitted version.

## Conflict of Interest

DS received personal fees from LEO Pharma, Bayer, BMS/Pfizer, Aspen, and CARMAT and received travel grant from Boerhinger Ingelheim. NG received personal fees from LEO Pharma, Bayer, BMS/Pfizer, Boerhinger Ingelheim, and Aspen and received travel grant from Boerhinger Ingelheim. The remaining authors declare that the research was conducted in the absence of any commercial or financial relationships that could be construed as a potential conflict of interest.

## Publisher's Note

All claims expressed in this article are solely those of the authors and do not necessarily represent those of their affiliated organizations, or those of the publisher, the editors and the reviewers. Any product that may be evaluated in this article, or claim that may be made by its manufacturer, is not guaranteed or endorsed by the publisher.
